# Experimental Ischemic Stroke–Induced Alpha-Synuclein Pathology Enhances Endothelial Inflammatory Response and Impairs Angiogenesis

**DOI:** 10.1161/STROKEAHA.125.052265

**Published:** 2025-09-18

**Authors:** Tizibt Ashine Bogale, Domenico Mercurio, Alessia Valente, Serena Seminara, Gaia Faustini, Francesca Longhena, Aurora Bianchi, Stefania Mitola, Arianna Bellucci, Stefano Fumagalli, Marina Pizzi

**Affiliations:** 1 Division of Pharmacology (T.A.B., G.F., F.L., S.M., A. Bellucci, M.P.), Department of Molecular and Translational Medicine, University of Brescia, Italy.; 2 Biotechnology Division (S.M.), Department of Molecular and Translational Medicine, University of Brescia, Italy.; 3 Department of Acute Brain and Cardiovascular Injury, Istituto di Ricerche Farmacologiche Mario Negri IRCCS, Milan, Italy (T.A.B., D.M., A.V., S.S., A. Bianchi, S.F.).

**Keywords:** alpha-synuclein, angiogenesis, inflammation, ischemia, stroke

## Abstract

**BACKGROUND:**

α-Syn (alpha-synuclein) increases and oligomerization contributes to ischemic brain damage. Nevertheless, the exact mechanisms of ischemia-induced α-Syn pathology after stroke are understudied. In this exploratory and hypothesis-testing study, we specifically investigated how ischemia-induced α-Syn pathology impacts vascular inflammation and angiogenesis.

**METHODS:**

Transient focal cerebral ischemia was induced in C57BL/6J wild-type and C57BL/6S mice with spontaneous deletion of α-Syn. In total, 72 mice were randomized to receive sham (12) or ischemia (60), with 1:1 allocation to genotype. Ischemic mice were included if presenting ≥3 intraischemic focal deficits. Experimental end points were 48 hours (acute phase) and 7 days (subacute phase) of reperfusion. Sensorimotor assessment, elevated plus maze, and survival analysis were stroke outcome readouts. Brain samples were collected for quantitative real-time polymerase chain reaction, Western blot, and immunohistochemistry. In vitro ischemia in brain microvascular endothelial cells was used to investigate the direct effect of extracellular α-Syn.

**RESULTS:**

In vivo, cerebral ischemia induced α-Syn 2.5-fold change gene expression, 2.7- to 3.2-fold-change protein oligomerization with apparent localization around cortical ischemic vessels. Improved subacute functional recovery linked to better survival was observed in C57BL/6S α-Syn null (87% to end point) versus C57BL/6J (45%) ischemic mice. The α-Syn null mice had reduced expression of *ICAM-1*, *MMP-9*, and vascular permeability factors, associated with less infiltrating leukocytes and juxta-vascular microglia in the acute phase. In the subacute phase, they showed upregulated proangiogenic factors (*VEGF-A*, *VEGFR-2*, *Angpt-2*, and *IL-6*) and angiogenic vessels. In vitro, brain microvascular endothelial cells upregulated *ICAM-1*, *IL-6*, and *VEGFR-2* expression when exposed to recombinant α-Syn at the beginning of ischemia and to a larger extent when α-Syn was preconditioned for 18 hours in hypoxia.

**CONCLUSIONS:**

These findings indicate that ischemia-triggered pathological α-Syn leads to worse outcome in stroke mice by promoting endothelial inflammatory response and immune cell infiltration during the acute phase and by limiting angiogenesis in the subacute phase.

Ischemic stroke is a devastating condition that affects millions of people worldwide and occurs due to occlusion of a brain artery, often resulting in neuronal cell loss. Moreover, early and progressive vascular dysfunction and damage can lead to blood-brain barrier leakage, cerebral edema, and hemorrhage, all contributing to poor outcomes in stroke patients.^1^ However, the reduction in blood flow caused by stroke can also trigger reparative processes, including vessel remodeling and angiogenesis, which are correlated with improved outcomes and better functional recovery.^2^ Only 3% to 8% of ischemic stroke patients are eligible for intravenous thrombolysis, with only 20% to 66% achieving complete recanalization.^3^ And in those who received these therapies, reperfusion of brain tissue downstream the recanalized artery is not guaranteed due to microvascular dysfunction and ongoing blood-brain barrier damage.^4^ Hence, therapies are needed to protect the brain from postrecanalization tissue damage, and to support de novo reparative processes, especially after the acute phase of ischemic stroke, when therapeutic options are scarce.

Recent evidence supports that α-Syn (alpha-synuclein), the protein forming insoluble fibrillary aggregates in the brain of patients affected by Parkinson disease (PD), Lewy body dementia, and multiple system atrophy, may be involved in poststroke brain injury.^5^ α-Syn is a small protein largely expressed by neurons and to a lesser extent by glia, blood cells, and vascular endothelial cells (ECs).^6–8^ The protein is also released extracellularly and can be found in body fluids.^9^ Following ischemic stroke, α-Syn is induced, oligomerized, and translocated into the nucleus, and its absence or suppression of its production protected rodents from progressive brain damage after experimental ischemic stroke.^5,10,11^ However, the exact mechanisms by which ischemia-induced α-Syn pathology contributes to brain damage or impaired recovery after stroke remain unknown. Previous findings indicate that toxic α-Syn species exert deleterious effects on ECs cultures by impairing both the expression of TJ (tight junction) proteins and exocytosis of vasoactive substances from Wiebel-Palade bodies.^12^ Moreover, a study in young transgenic mouse model expressing familial PD-associated A53T mutant α-Syn showed that the accumulation of toxic α-Syn is paralleled by reduction of EC proliferation in the absence of vascular cell death or neuroinflammation.^13^

We thus hypothesized that ischemia-altered α-Syn exacerbates brain damage by affecting cerebrovascular inflammation during the acute phase (48 hours after transient middle cerebral artery occlusion [tMCAo]) or angiogenesis in the subacute phase (7 days after tMCAo). To test this hypothesis, 60 min middle cerebral artery occlusion in wild-type (C57BL/6J) mice and in mice with a spontaneous deletion of α-Syn gene (C57BL/6S α-Syn Null)^14,15^ was performed by unilateral intraluminal filament insertion. Mice were assessed for sensorimotor deficits, anxiety-like phenotype, general locomotor function, and survival as stroke outcomes. At 48 hours and 7 days after reperfusion, brains were evaluated for α-Syn expression, localization, immune cell infiltration, perivascular microglia, angiogenesis, and TJ protein levels using immunohistochemistry, real-time polymerase chain reaction, and Western blot. Moreover, to dissect the direct effect of α-Syn in ischemic vasculature, microvascular ECs were subjected to oxygen-glucose deprivation and reoxygenation injury in the presence or absence of naïve or hypoxia-preconditioned α-Syn. Then gene expression for the marker of endothelial activation and proinflammatory cytokine were measured. In addition, the uptake and accumulation of exogenous α-Syn in ECs was evaluated by immunofluorescence.

## Methods

### Availability of Data and Materials

All data will be publicly available at this online repository on Figshare: doi:10.6084/m9.figshare.28164035.

Please see the Major Resources Table in the Supplemental Material.

### Mouse Experiments

All animal experiments were approved by ethical committee at the Istituto di Ricerche Farmacologiche Mario Negri IRCCS (Istituto di Ricovero e Cura a Carattere Scientifico) and by the Italian Ministry of Health (authorization number n°383/2021-PR). Male 9-to-12 week old C57BL/6J wild-type (Charles Rivers-Italy) and C57BL/6JOlaHsd (C57BL/6S, Envigo/Inovit) were used. The C57BL/6S mice are subpopulations of C57BL/6J inbred mice that exhibit a spontaneous deletion of the *SNCA* and *Mnrn1* gene locus.^14,15^ All the protocols and details of this study are in accordance with the ARRIVE guidelines (Animal Research: Reporting of In Vivo Experiments).^16^ We used only male mice because estrogen affects ischemic outcome in experimental models, which could interfere with interpretation of the results.^17^

### Experimental Design

Mice were randomly assigned to experimental groups, and analyses were run in a blinded fashion. Given the explorative nature of our research, the power test study considered a decrease of 30% of neuroscore at 48 hours as an index of ameliorated sensorimotor deficits. Group size was defined pre hoc using: *n*=2σ^2^f (α,β)/Δ^2^ (SD in groups=σ, type 1 error α=0.05, type II error β=0.2, percentage difference between groups Δ=30). The SD between groups was calculated based on previous neuroscore data with the model (σ=25, yielding *n*=10.9). At each experimental end point, the mice were divided into 2 groups for tissue collection, facilitating cross-sectional histological or molecular analysis.

### Focal Cerebral Ischemia

Mice underwent intraluminal occlusion of the right middle cerebral artery for 60 minutes (tMCAo) as described previously.^18^

Ischemic mice were included if presenting ≥3 of the following intraischemic deficits:

1. The palpebral fissure had an ellipsoidal shape (not the normal circular one).
2. One or both ears extended laterally.
3. Asymmetrical body bending on the ischemic side.
4. Limbs extended laterally and did not align with the body.

Excluded criteria for mice included the following:

1. They died during middle cerebral artery surgery.
2. There was a major surgical violation.
3. They had a decrease in body weight of <35% before sacrifice compared with baseline.

Total of 72 mice (12 sham-operated and 60 ischemic mice) were used in this study, including 1 excluded mouse per exclusion criteria.

### Health Monitoring

Over the 48 hours after surgery, mice were monitored in accordance with the IMPROVE guidelines (Ischaemia Models: Procedural Refinements Of in Vivo Experiments)^19^ to opt for analgesia (0.05–0.1 mg/kg Buprenorphine subcutaneously) or for humane end point in case of moderate to severe distress. Mice euthanized before end point received the worst behavioral score.

### Behavioral Deficits

Sensorimotor deficits were assessed by the neuroscore as previously described.^20^

### Elevated Plus Maze

The elevated plus maze was used to evaluate anxiety-like behavior and was done as previously described.^21^

### Tissue Processing

For histology, brains were harvested after transcardiac perfusion with 30 mL of PBS 0.1 mol/L, pH 7.4, followed by 60 mL of chilled paraformaldehyde (4%) in PBS, sucrose-sink, and frozen. For molecular biology, brains were harvested and snap-frozen.

### Immunohistochemistry

Cryostate-cut 20 µm-thick sections were stained using the anti-CD45 (cluster of differentiation 45 [leukocyte marker]) antibody (no. 553078; BD Biosciences) followed by reaction with 3,3-diaminobenzidine tetrahydrochloride (Vector Laboratories, CA). Images were captured on an Olympus BX61 Virtual Stage microscope at ×20 magnification. CD45-positive cells were counted using the ImageJ cell counter and expressed as density per square millimeter.

### Immunofluorescence

Cryostate-cut 20 µm-thick sections were stained using the anti–α-Syn (no. 610787; BD Biosciences), anti-CD31 (cluster of differentiation 31 [endothelial marker], no. 550274; BD Biosciences), anti–PSD-95 (postsynaptic density protein 95, no. ab18258; Abcam), and anti-Ki67 (no. 9129; Cell Signaling) antibodies. Then, sections were incubated with secondary antibody conjugated to Alexa-fluor488, 647, or 555. For the in vitro experiment, cells were washed with PBS and fixed in 4% paraformaldehyde. Immunofluorescence images were acquired at ×40 or ×60 magnification by a Nikon A1 confocal system and captured using NIS (Nikon Imaging Software)-Elements software. Images were then reconstructed using Imaris software.

### Western Blot Analysis

Total proteins from brain tissues were extracted in homogenization buffer with a complete protease and phosphatase inhibitor mixture. Proteins (15–30μg) were run on 8% to 12% polyacrylamide gels, transferred onto a polyvinylidene fluoride membrane, and probed with anti-mouse α-Syn (no. 610787; BD Biosciences), anti-mouse actin (MAB1501; Merck), anti-occludin (no. 71-1500; Thermo Fisher), and anti-zonulin1 (no. 33-9100; Thermo Fisher) antibodies. Secondary antibodies were goat anti-mouse and anti-rabbit HRP (horseradish peroxidase). Densitometry analysis of the bands was performed using ImageJ after normalization to actin.

### In Vitro Ischemia

The immortalized human brain microvascular EC cell line was grown in fibronectin-coated ibidi 96-well micro plates to allow adhesion and growth with molecular and cellular developmental biology medium (Biowest serum-reduced supplementation medium) supplemented with 0.1% hydrocortisone and 1% EC growth factor, FBS 5%, l-glutamine 1%, penicillin/streptomycin 1% at 37 °C in a humidified incubator with 5% CO_2_. To induce oxygen-glucose deprivation, plates were bathed with DMEM, no glucose, no glutamine, and no FBS in a hypoxic chamber (99% nitrogen and <1% oxygen) at 37 °C for 2 hours. For reoxygenation, half medium was changed to low-glucose DMEM and transferred to a humidified incubator with 5% CO_2_ for 4 hours with nonhypoxia preconditioned or hypoxia preconditioned 6 µg/mL (0.415 µM) endotoxin-free monomers of human recombinant α-Syn. At the end of the experiment, cells were scraped off for subsequent RNA extraction (see below) or paraformaldehyde-fixed for IF. For the latter case, cells were stained using anti–α-Syn and Phalloidin-fluor-488 (no. ab176753; Abcam).

### Quantitative Real-Time Polymerase Chain Reaction

RNA was extracted from ipsilateral hemispheres using the RNeasy Mini Kit for total RNA extractions (PureLink RNA Mini Kit; Thermo Fisher). Then, 1 μg of total RNA was retro transcribed to cDNA, amplified by real-time polymerase chain reaction (using Power SYBR Green, Applied Biosystems). Relative gene expression was determined with the ΔΔCt (Delta Delta cycle threshold) method (β-actin as housekeeping gene). Primer sequences are detailed in Supplemental Material.

### Statistics

Group comparisons were conducted using *t* tests, Mann-Whitney *U* test tests, or relevant 2-way ANOVA followed by the appropriate post hoc test. Bartlett test checked equal variances and, if not equal, a Welch correction was applied to the test. Statistical analyses were performed using GraphPad Prism (version 9.0; GraphPad Software Inc, San Diego, CA). All data were presented as mean and SD. *P*<0.05 was considered statistically significant.

## Results

### α-Syn Expression, Oligomerization, and Association With Cortical Vessels After Ischemic Stroke

**Figure 1.**
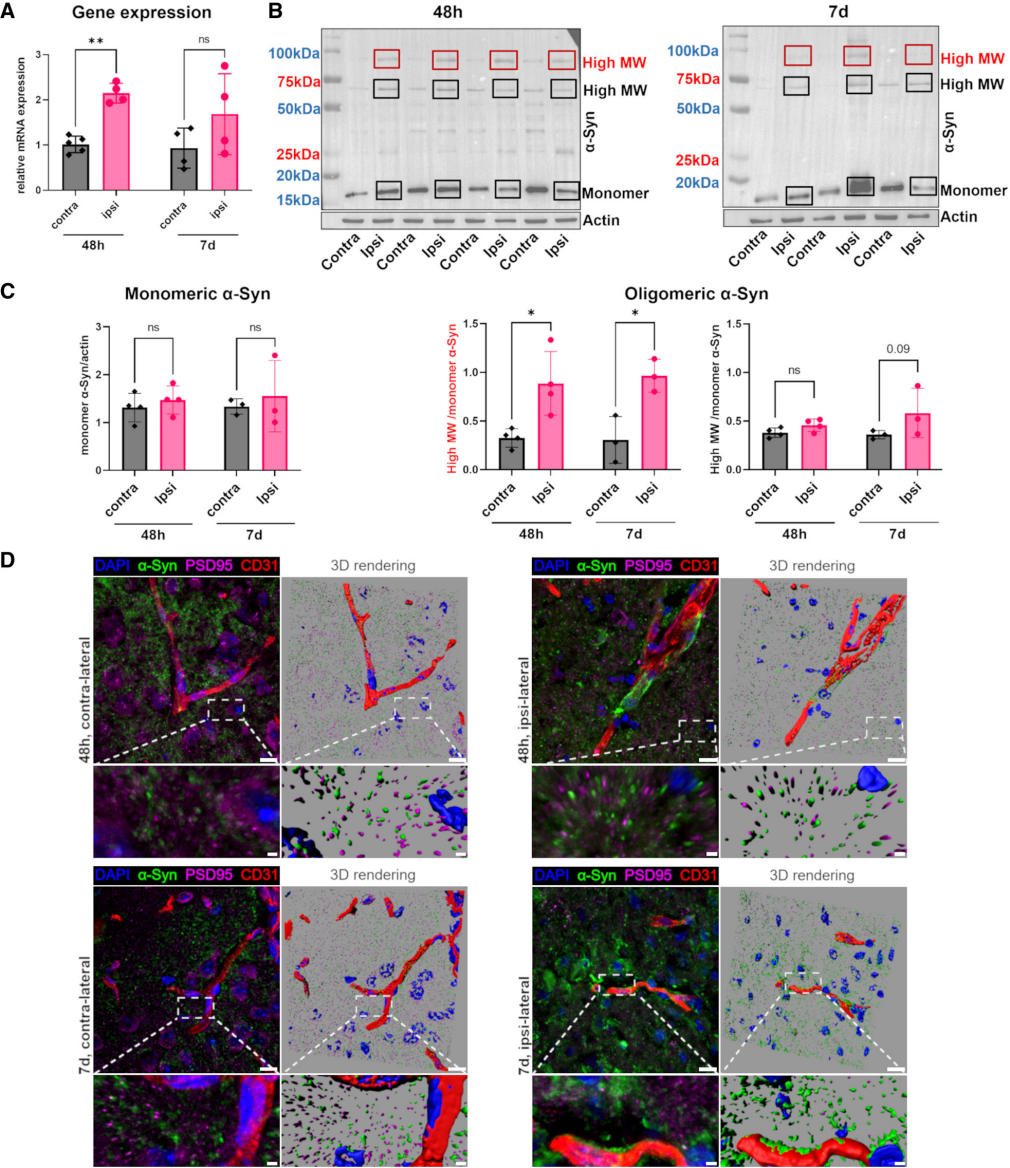
**α-Syn (alpha-synuclein) mRNA increase, oligomerization, and deposition on cortical vessels after stroke. A**, Relative expression of *SNCA* in ipsilateral cortex of transient middle cerebral artery occlusion (tMCAo) mouse 48 hours and 7 days after cerebral ischemia compared with contralateral cortex (n=4). **B**, Western blotting for α-Syn and β-actin from contralateral (Contra) and ipsilateral (Ipsi) cortex of wild-type mice. **C**, Densitometric quantification of Western blot images in **C** for α-Syn monomeric and oligomeric protein levels 48 hours (n=4) and 7 days (n=3) after ischemic stroke. **D**, Immunofluorescence microphotographs of ischemic and contralateral cortex of C57BL/6J mice labeled for α-Syn (green), PSD-95 (postsynaptic density protein 95; purple), CD31 (cluster of differentiation 31 [endothelial marker]; red), and nuclei (blue) at 48 hours and 7 days after tMCAo. Scale bars 10 µm in the **top**, 2 µm in the **bottom**, magnified. Data are presented as individual values ± SD and analyzed by 2-way ANOVA multiple comparisons followed by Sidak post hoc test,*P<0.05, **P<0.01. 3D indicates 3-dimensional; DAPI, 4′,6-diamidino-2-phenylindole; MW, molecular weight; and ns, not significant.

First, we assessed α-Syn mRNA expression after tMCAo and 48 hours of reperfusion. We found a significant increase in α-Syn mRNA expression in the cortex ipsilateral to tMCAo versus the contralateral area (Figure 1A). Interestingly, Western blot analysis comparing the levels of total and oligomeric α-Syn in cortex ipsilateral and contralateral to tMCAo, showed that although the total levels of α-Syn were not different between nonischemic and ischemic cortices, the latter exhibited a significant accumulation of oligomeric α-Syn species (100 kDa, red box). Interestingly, the elevation of oligomeric α-Syn was sustained even at 7 days after tMCAo (Figure 1B and 1C). To understand the cellular localization of α-Syn after tMCAo, we performed α-Syn, PSD-95 (a postsynaptic marker), CD31 (vessel marker) immunolabeling plus DAPI (4′,6-diamidino-2-phenylindole)–based nuclear staining on brain sections, which were then analyzed by confocal microscopy (Figure 1D). We found that in contralateral cortices, α-Syn was localized at presynaptic terminals, as supported by the localization in proximity to the postsynaptic marker PSD-95 (Figure 1D). Interestingly, in the tMCAo side (Figure 1D, 3-dimensional renderings), α-Syn was found to progressively accumulate inside perivascular cells at 48 hours and on blood vessels at 7 days (Figure 1D). At this point, PSD-95–positive puncta appeared to decrease in the ipsilateral side, compatible with the occurrence of synaptic degeneration and loss.

### α-Syn Null Mice Exhibit Improved Functional Recovery From Ischemic Stroke

**Figure 2.**
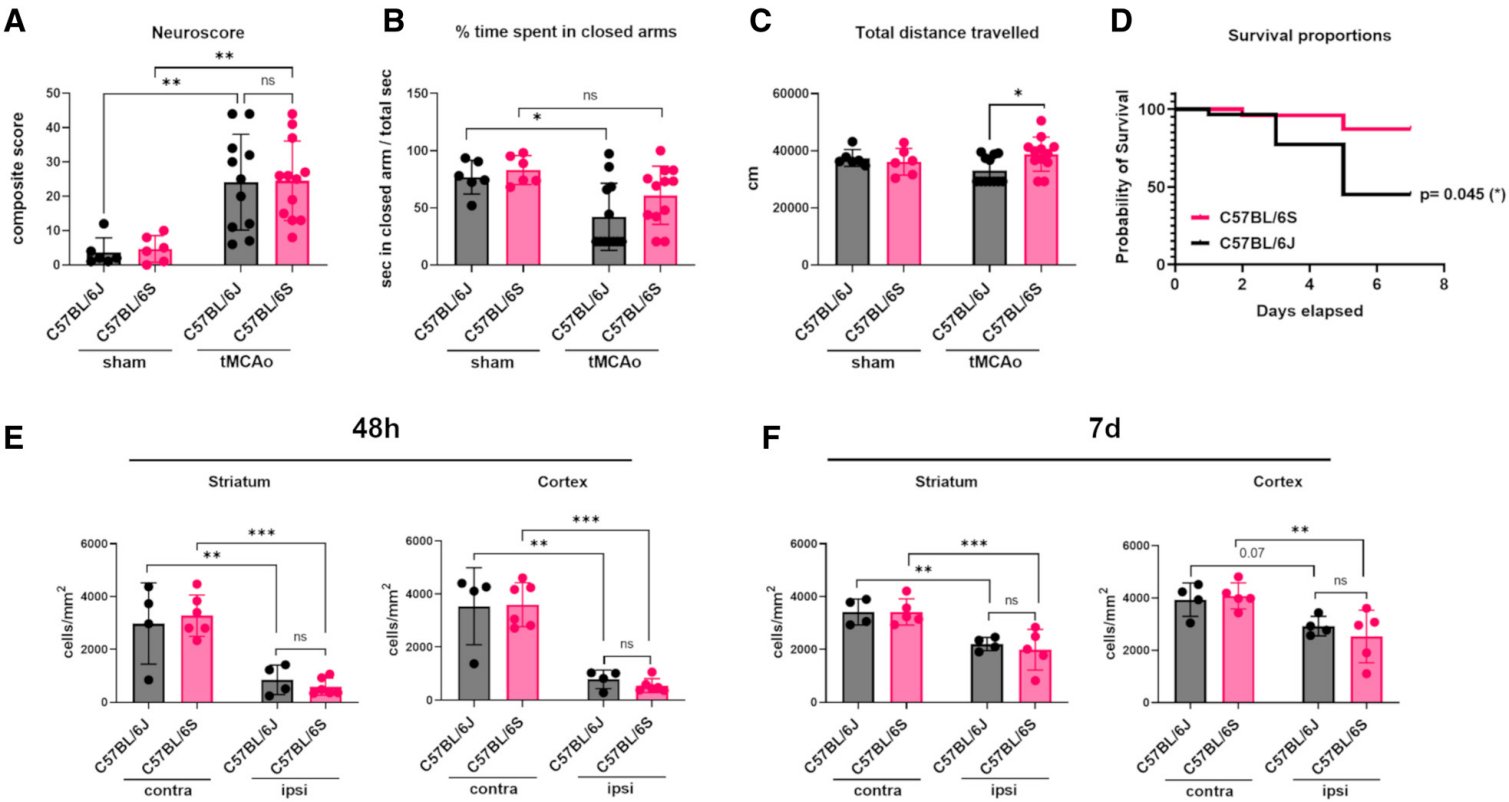
**α-Syn (alpha-synuclein) deficiency facilitates recovery from ischemic stroke. A**, Sensorimotor deficits assessed by using a composite neurological score 48 hours after transient middle cerebral artery occlusion (tMCAo; n, sham=6; tMCAo=11 for C57BL/6J, 12 for C57BL/6S). **B**, The ratio of time spent in closed arms to the total time (measured in seconds) spent in both open and closed arms assessed using elevated plus maze (EPM) 7 days after tMCAo (n=6 for sham, n=12 for tMCAo). **C**, The total distance traveled in the open arms, closed arms, and center measured in centimeters using EPM 7 days after tMCAo (n=6 for sham, n=12 for tMCAo). **D**, The Kaplan-Meier survival curve showing better survival in C57BL/6S than C57BL/6J mice in the days after 60 min tMCAo (Log-rank Mantel-Cox test, *P*=0.045, n=29 for C57BL/6J and 24 for C57BL/6S). **E** and **F**, Quantification of neuronal density in the ipsilateral (Ipsi) and contralateral (Contra) striatum and cortex was done on cresyl-violet stained brain sections 48 hours (n=4 for C57BL/6J, n=6 for α-Syn Null) and 7 days (n=4 for C57BL/6J, n=5 for C57BL/6S) after tMCAo. Data are presented as mean±SD and analyzed by 2-way ANOVA followed by Sidak post hoc test,*P<0.05, **P<0.01, ***P<0.001, ns=not significant.

To assess whether and how α-Syn plays a key role in poststroke functional recovery, we subjected C57BL/6S α-Syn null mice and C57BL/6J control mice to ischemia-reperfusion injury and then measured neurological deficits, anxiety-like phenotype, locomotor function, survival, and neuronal loss as measures of stroke outcomes. Sensorimotor deficits were assessed 48 hours after tMCAo using a composite neuroscore as previously described.^20^ Ischemia induced comparable deficits in C57BL/6J and C57BL/6S mice during the acute phase of tMCAo (48 hours, Figure 2A). General locomotor activity and anxiety-like phenotype were assessed during the subacute phase (7 days) using the elevated plus maze test. The results showed that, in comparison to C57BL/6J ischemic mice, C57BL/6S tMCAo mice spent more time in closed arms similar to sham controls, and traveled more distance throughout the elevated plus maze platform, suggesting an amelioration of ischemia-induced disinhibited behavior and an improved recovery of locomotor function in these latter mice (Figure 2B and 2C). Seven days after tMCAo, the survival rate of C57BL/6S α-Syn null mice was significantly higher (87%) than that of C57BL/6J mice (45%, Figure 2D), with all the observed deaths occurring within 5 days after tMCAo. Quantification of neuronal loss on cresyl-violet stained brain sections revealed a significant neuronal loss in the ipsilateral striatum (71% in C57BL/6J versus 82% in C57BL/6S at 48 hours and 35% in C57BL/6J versus 42% in C57BL/6S at 7 days) and ipsilateral cortex (77% in C57BL/6J versus 84% in C57BL/6S at 48 hours and 25% in C57BL/6J versus 39% in C57BL/6S at 7 days) with no significant differences between the genotypes (Figure 2E and 2F).

### Ischemia-Induced Upregulation of Key Genes Involved in Endothelial Activation, Immune Cell Infiltration, and Basement Membrane Degradation Is Reduced in the Absence of α-Syn 48 Hours After tMCAo

**Figure 3.**
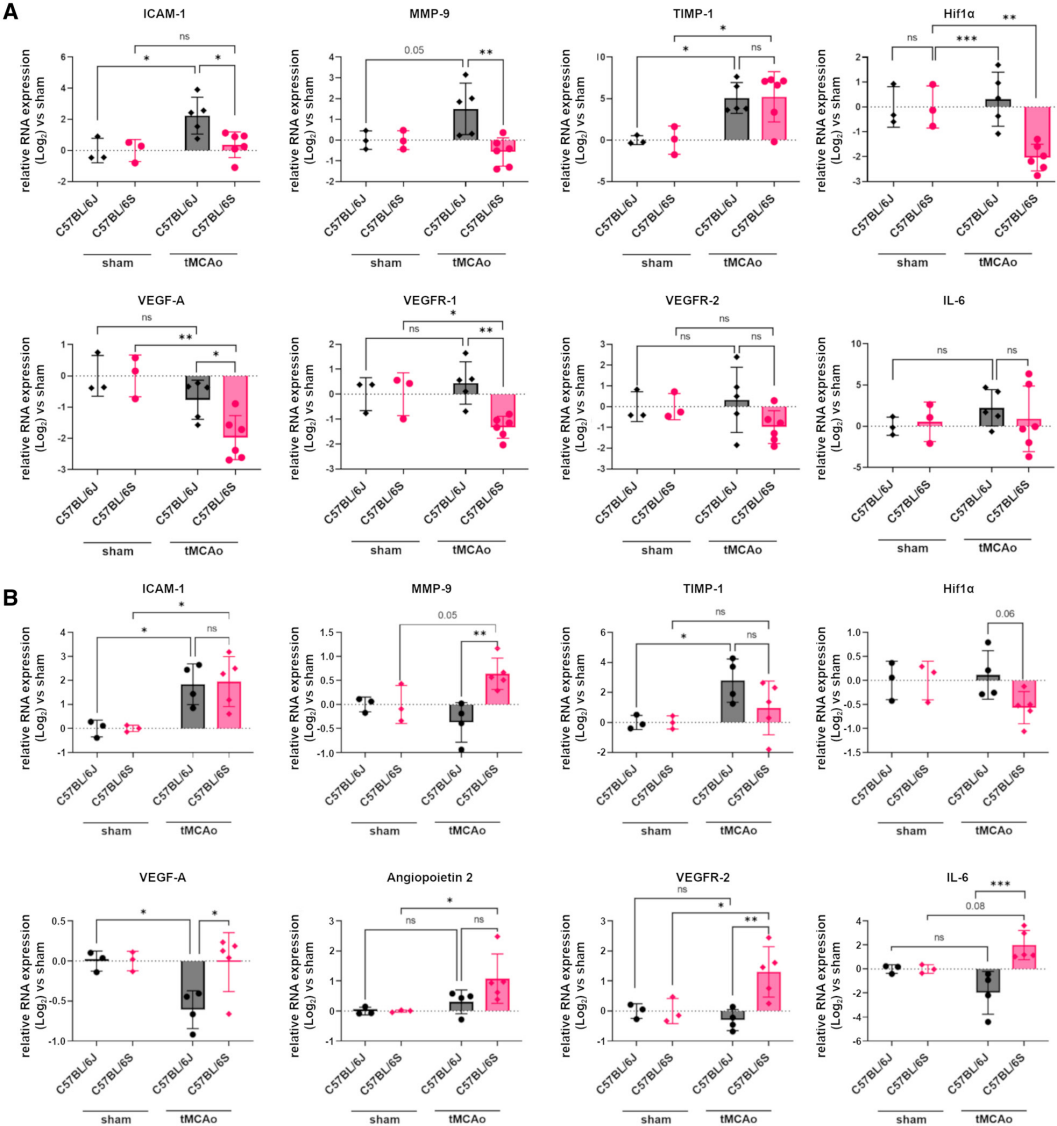
**α-Syn (alpha-synuclein) absence modulates the expression of vessel-associated genes after transient middle cerebral artery occlusion (tMCAo).** Real-time quantitative polymerase chain reaction was performed using total RNA extracted from ischemic brains 48 hours and 7 days after tMCAo (n=4 for C57BL/6J and n=5–6 for C57BL/6S) or from sham-operated brains (n=3).The mRNA expression at 48 hours (**A**) and (**B**) at 7 days. Data are presented as individual values ±SD and analyzed by 2-way ANOVA followed by Sidak post hoc test,*P<0.05, **P<0.01, ***P<0.001, ns indicates not significant.

To probe the impact of postischemic increase of α-Syn expression and perivascular localization, we assessed the expression of *ICAM-1* (intercellular adhesion molecule-1), *MMP-9* (matrix metallopeptidase 9), *VEGF-A* (vascular endothelial growth factor A), and *VEGFR-1* (vascular endothelial growth factor receptor 1), which are key genes involved in endothelial activation, immune cell infiltration, basement membrane degradation and vascular permeability after ischemic stroke. We found that the gene expression of *ICAM-1* and *MMP-9* was lower in C57BL/6S α-Syn null compared with C57BL/6J mice at 48 hours after tMCAo (Figure 3A). Although gene expression of *VEGF-A* was not altered by ischemia in C57BL/6J mice 48 hours after reperfusion, it was significantly suppressed in C57BL/6S α-Syn null mice (Figure 3A). The mRNA levels of *VEGFR-1* were downregulated in ischemic C57BL/6S α-Syn null, but not in C57BL/6J mice (48 hours; Figure 3A). These results suggest a role of α-Syn in the activation of early ischemic vascular signaling pathways.

### Mice Lacking α-Syn Expressed Higher Levels of Proangiogenic Factors 7 Days After tMCAo

At 7 days from tMCAo, we found that C57BL/6S α-Syn null mice exhibited a significant increase in the expression of *VEGF-A*, *VEGFR-2*, angiopoietin-2, *MMP-9*, and *IL-6* (interleukin 6) in ipsilateral cortices compared with ischemic C57BL/6J mice (Figure 3B), suggesting a role of ischemia-induced α-Syn in limiting vascular reparative processes.

In mice lacking α-Syn, the mRNA levels of *HIF1α* were significantly reduced after brain ischemia, especially at 48 hours after reperfusion, compared with wild-type C57BL/6J mice (Figure 3A and 3B). During the acute phase, TIMP (tissue inhibitor of metalloproteinase) 1 (*TIMP-1*) gene expression levels were significantly upregulated in both C57BL/6J and C57BL/6S α-Syn null ischemic cortices compared with their respective sham controls (Figure 3A). However, in the subacute phase, *TIMP-1* levels became comparable between sham and C57BL/6S tMCAo mice while levels remained elevated in C57BL/6J mice at 7 days after reperfusion (Figure 3B). Finally, *ICAM-1* mRNA appeared significantly more elevated either in C57BL/6S α-Syn null mice or in C57BL/6J when compared with their respective sham genotypes after 7 days of reperfusion (Figure 3B). In summary, the absence of α-Syn alters the expression dynamics of key genes involved in the ischemic response, such as *HIF-1*α** and its downstream target genes (*VEGF-A*, and *VEGFR-1*) after brain ischemia. While the expression of *TIMP-1* in the acute phase seems to be independent of α-Syn, the delayed increase in *ICAM-1* in null mice during subacute phase may indicate an activation of α-Syn–independent alternative or compensatory inflammatory pathways.

### α-Syn Absence Reduced Immune Cell Infiltration and Microglia-Vascular Contacts After Focal Cerebral Ischemia

Because the above-presented results support that α-Syn plays a role in regulating key factors linked to leukocyte recruitment into the brain (ICAM-1 and MMP-9), we further investigated whether α-Syn impacts the infiltration of immune cells after stroke. Considering that microglia are a significant source of MMP-9 after focal brain ischemia,^22^ and their association with blood vessels can contribute to vascular disintegration,^23^ we additionally explored microglia-vascular contacts in this context.

**Figure 4.**
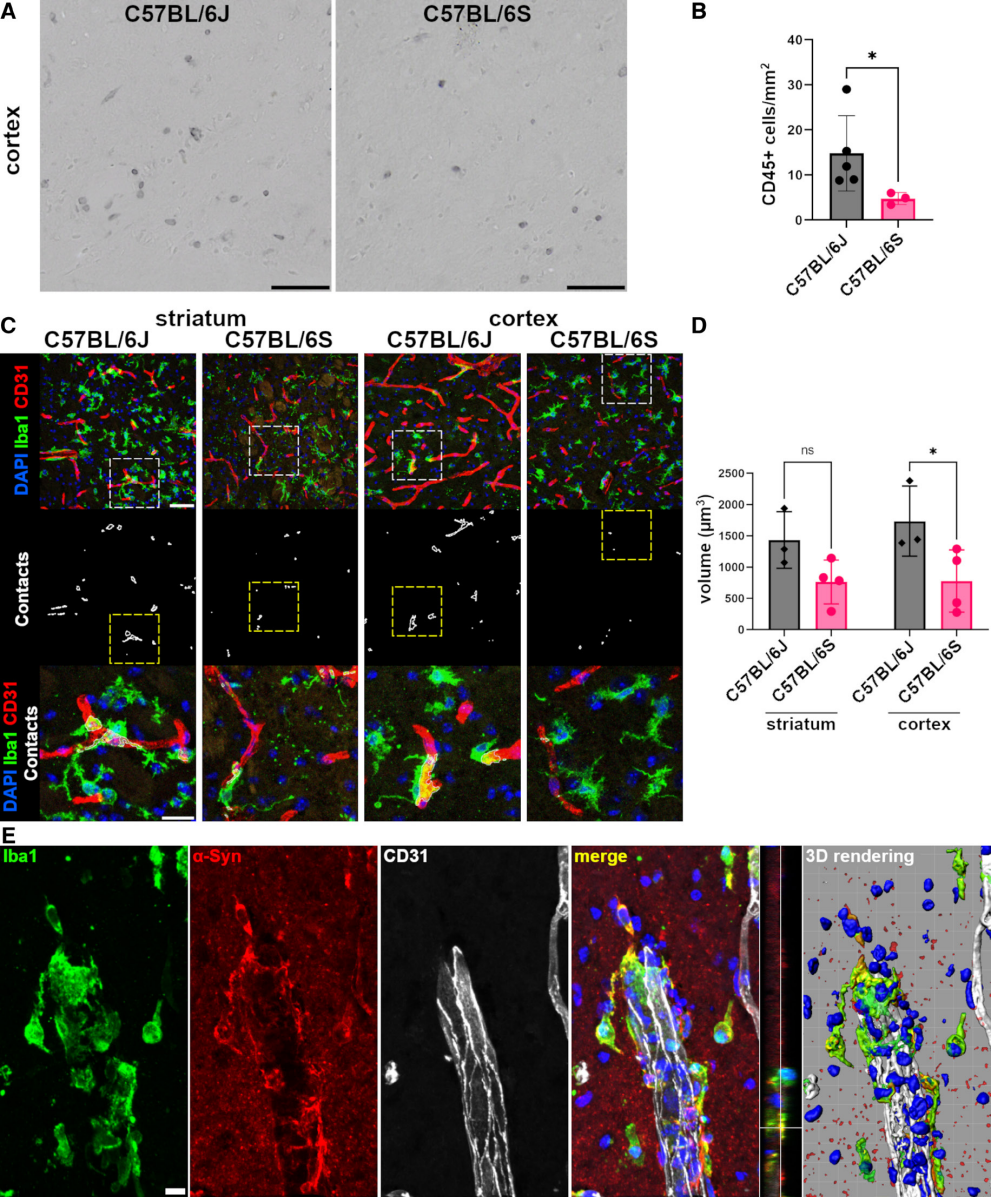
**α-Syn (alpha-synuclein) absence attenuates early leukocyte infiltration and vessel contacts by microglia during the acute phase of ischemic stroke. A**, Representative brightfield microphotographs of CD45 (cluster of differentiation 45 [leukocyte marker]) staining in ischemic cortex of C57BL/6J and C57BL/6S mice 48 hours after transient middle cerebral artery occlusion (tMCAo). Scale bars 50 µm. **B**, Quantification of the density of CD45+ leukocytes per mm^2^ in ipsilateral cortex of C57BL/6J (n=5) and C57BL/6S (n=3) mice. Data are presented as individual values±SD and analyzed by Unpaired *t* test,*P<0.05. **C**, Representative immunofluorescence microphotographs of striatum and cortex labeled for CD31 (cluster of differentiation 31 [endothelial marker]; red), Iba1 (ionized calcium-binding adapter molecule 1 [microglia marker], green), nuclei (labeled with DAPI [4′,6-diamidino-2-phenylindole], blue) at 48 hours after tMCAo, scale bars 50 µm. The white outlines show segmented contacts that are magnified in **lower bottom. D**, Quantification of vascular Iba1 volume in cubic millimeters showed a decrease in C57BL/6S (n=4) ischemic cortex at 48 hours tMCAo in comparison to C57BL/6J mice (n=3). Data are presented as individual values±SD and analyzed by 2-way ANOVA followed by Sidak post hoc test,*P<0.05, ns=not significant. **E**, Representative immunofluorescence microphotographs of cortex of C57BL/6J labeled for Iba1 (green), α-Syn (red), CD31 (white), and nuclei (labeled with DAPI, blue) at 48 hours after tMCAo, scale bar 10 µm. The 3-dimensional (3D) rendering presents the segmented isosurfaces of all channels and includes the Iba1/α-Syn merged signal (yellow).

We observed that CD45-immunoreactive leukocytes were detected in ischemic cortices 48 hours after tMCAo (Figure 4A). CD45-positive cell quantification showed that C57BL/6S α-Syn Null mice had marked reduction in the number of recruited leukocytes in ipsilateral cortex versus wild-type mice (Figure 4B).

Supporting a less inflamed microenvironment in α-Syn Null mice in light of the reduced expression of proinflammatory and vascular homeostatic factors and leukocyte infiltration after tMCAo, we thus assessed microglia-vascular contacts in Iba-1 and CD31 immunolabeled sections containing striatal and cortical areas. Results from the quantification of Iba1-positive microglia and CD31 immunopositive vessels (Figure 4C and 4D) showed a decrease in the vascular volume contacted by microglia branches in the ipsilateral striatum and cortex of C57BL/6S α-Syn null mice compared with C57BL/6J mice at 48 hours after reperfusion (Figure 4D). Of note, microglia contacting the brain vessels in the C57BL/6J ischemic mice showed colocalization with α-Syn at 48 hours after tMCAo (Figure 4E).

### α-Syn Null Mice Showed Augmented Ischemia-Induced Angiogenesis and Vascular Remodeling

**Figure 5.**
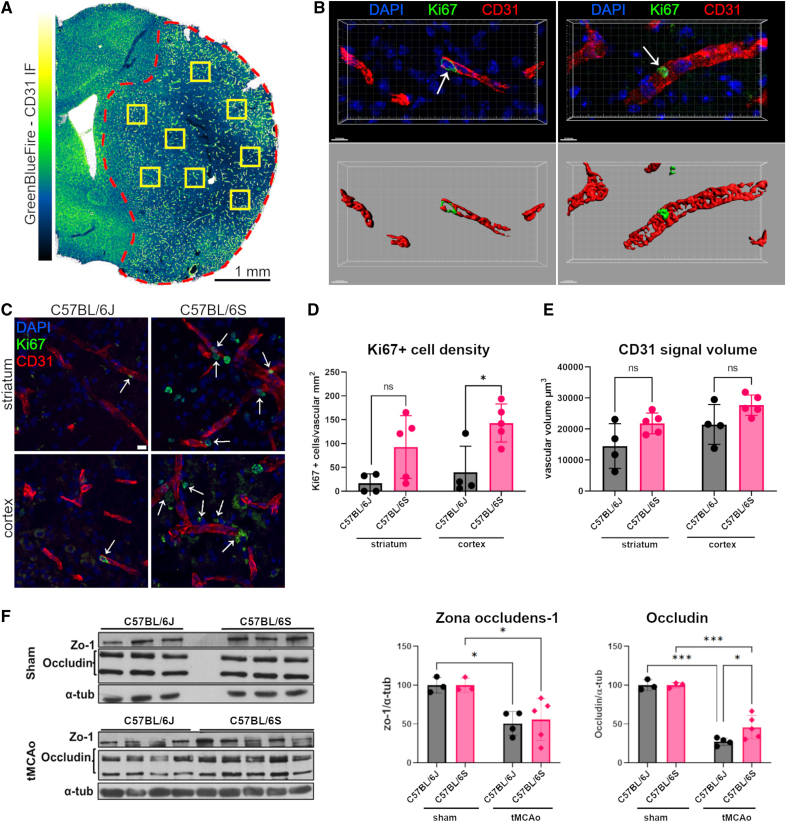
**Absence of α-Syn (alpha-synuclein) improves angiogenic processes. A**, Representative immunofluorescence microphotograph of the brain hemisphere labeled for CD31 (cluster of differentiation 31 [endothelial marker]; presented as GreenBlueFire color-coding). The ischemic region is demarcated by the dashed red line and contains the yellow boxes indicating the 8 fields of view (sized 320x320x10µm) acquired and analyzed. **B**, Representative 3D renderings of vessels (CD31, red) and duplicating cells (Ki67, green) in C57BL/6J (**left**) or C57BL/6S α-Syn Null (**right**) mice. The arrows indicate the Ki67+ cells integrated in the vasculature that were quantified to assess angiogenesis. **C**, Representative immunofluorescence microphotographs of striatum and cortex labeled for CD31 and Ki67, showing increased vessel-integrated Ki67 positive cells (arrows) in α-Syn Null mice, scale bar 10 µm. **D**, The quantification showed an increase in proliferating endothelial cells (CD31/Ki67+) in α-Syn Null mice (n=5) ipsilateral striatum and cortex compared with C57BL/6J mice (n=4). **E**, Analysis of the vascular volume (expressed in µm^3^) revealed an increasing trend in CD31 volume occupancy in α-Syn Null mice ipsilateral cortex and striatum compared with wild-type (WT) mice. **F**, Western blotting and densitometric analysis for Zo-1 and occludin in sham (n=3) and transient middle cerebral artery occlusion (tMCAo) cortex, C57BL/6J (n=4) compared with α-Syn Null (5) mice normalized to their respective sham controls. Data are presented as individual values±SD and analyzed by 2-way ANOVA followed by Sidak’s post hoc test,*P<0.05, **P<0.01, ***P<0.001. α-tub indicates alpha-tubulin; DAPI, 4′,6-diamidino-2-phenylindole; ns, not significant; and ZO-1, zonula occludens-1.

We then aimed to assess whether α-Syn was also involved in poststroke angiogenic process by performing double immunolabeling of CD31 and Ki67, a key cell proliferation marker. We sampled the tissue as depicted in Figure 5A, namely by a stereological placing of 4 high-resolution fields of view in the ipsilateral striatum and cortex. We identified and counted the Ki67-positive cells present within the CD31 signal, as shown in the 3-dimensional renderings in Figure 5B. Upon quantification, the density of Ki67-positive cells per CD31-labeled vessel area (in mm^2^) was increased in the ipsilateral striatum and, to a higher extent, in the cortex of C57BL/6S α-Syn null mice compared with C57BL/6J at 7 days after tMCAo (Figure 5C and 5D). α-Syn null mice displayed an augmentation, although not statistically significant, of CD31-positive signal volume in the ischemic striatum and cortex at day 7 compared with C57BL/6J mice, further supporting that angiogenic processes are increased in these mice (Figure 5E). To exclude that the Ki67 signal indicated proliferating immune cells, we double immunolabeled for Ki67 and CD45, the latter highly expressed by infiltrating leukocytes and, to a lesser extent, by microglia.^24^ As shown in Figure S1, CD45-positive cells did not co-localize with Ki67 in the ischemic cortex at 7 days after tMCAo.

Previous studies have shown that TJ-related proteins are elevated in the peri-infarct region after stroke and correlate directly with improved angiogenesis.^25^ We thus also assessed occludin and ZO-1 (zonula occludens-1) levels in the cortex of C57BL/6S α-Syn null and C57BL/6J mice 7 days after tMCAo by using western blot. In line with the above-reported evidence supporting an increase of angiogenesis in α-Syn null mice after stroke, we found that these animals exhibited significantly higher levels of occludin and to a lesser extent ZO-1 in the cortex ipsilateral to tMCAo when compared with C57BL/6J mice (Figure 5F).

### α-Syn Alteration in Hypoxic Environment Exacerbated I/R Injury in Microvascular ECs

**Figure 6.**
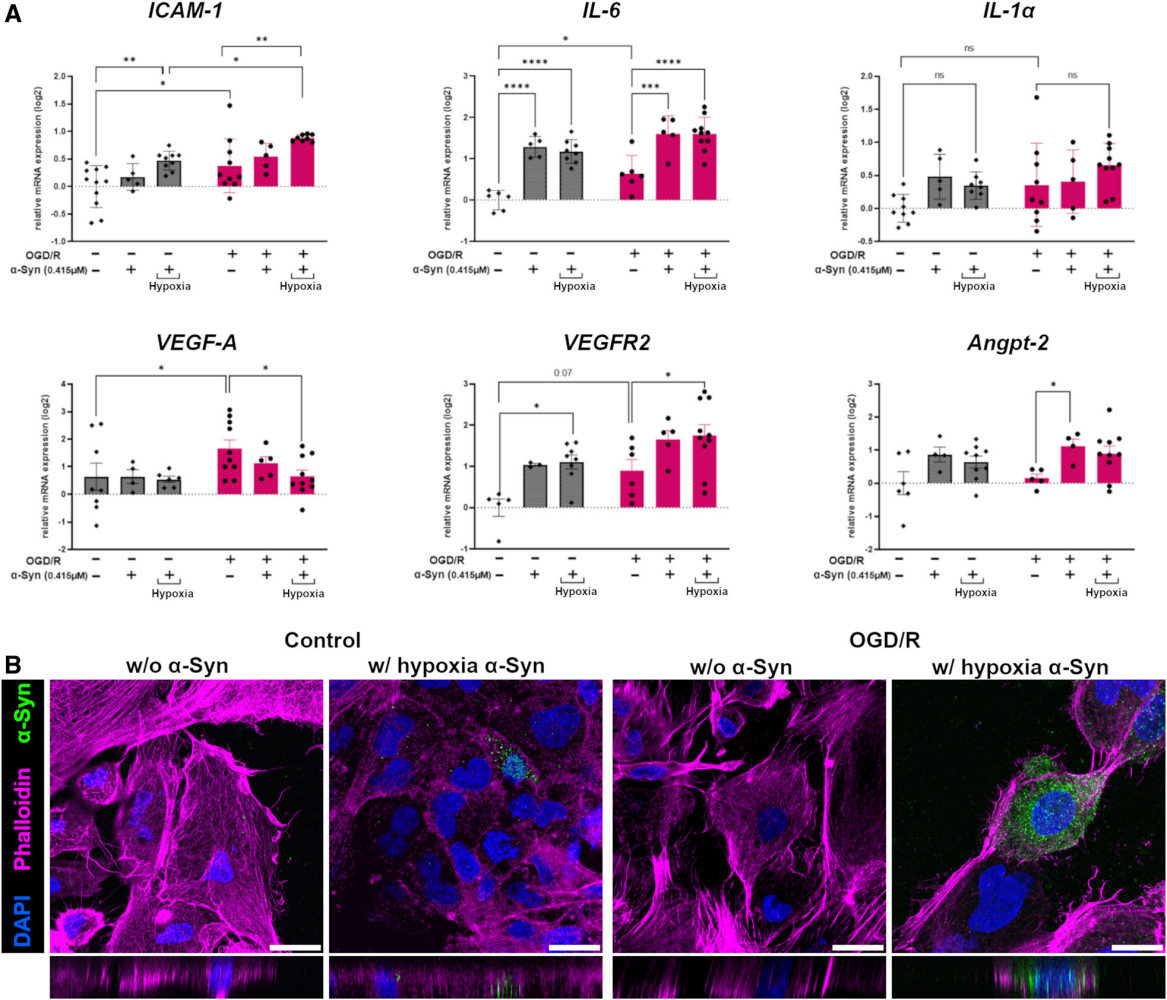
**Hypoxia-preconditioned α-Syn (alpha-synuclein) exacerbates ischemic injury in microvascular endothelial cells. A**, The gene expression of *ICAM-1*, *IL-6*, *Il-1a*, *VEGF-A*, *VEGFR-2*, and *Angpt-2* are shown respectively in ischemic immortalized human brain microvascular endothelial cells (ihBMECs; pink bars) vs control cells (gray bars) in the presence or absence of recombinant monomeric or hypoxia-preconditioned (hypoxia) α-Syn. Data are presented as individual values±SD and analyzed by 2-way ANOVA followed by Sidak post hoc test,*P<0.05, **P<0.01, ***P<0.001, ns=not significant. **B**, Representative immunofluorescence microphotographs of cultured ihBMECs labeled for α-Syn (green), phalloidin (purple), and nuclei (blue), showing α-Syn signal when oxygen-glucose deprivation and reoxygenation (OGD/R) cells were exposed to hypoxia-preconditioned α-Syn. Scale bars 20 µm. DAPI indicates 4′,6-diamidino-2-phenylindole; and ns, not significant.

To investigate how α-Syn might directly impinge on poststroke vascular dysfunction, we exposed immortalized human brain microvascular ECs to 2 hours of oxygen-glucose deprivation and 4 hours reoxygenation and measured the gene expression of *ICAM-1*, *IL-6*, *IL-1a*, *VEGF-A*, *VEGFR-2*, and *Angpt-2* (experimental plan shown in Figure S2). Two hours of oxygen-glucose deprivation followed by 4 hours reoxygenation significantly induced *ICAM-1* and *IL-6* compared with cells in the control condition. Because most of the α-Syn in the brain originates from neurons and has already undergone changes such as oligomerization, at the beginning of oxygen-glucose deprivation, we added naïve (unconditioned) or hypoxia-preconditioned endotoxin-free monomeric α-Syn at a concentration of 0.415µM (decided based on a dose-response curve, see Figure S3). Our result showed that *ICAM-1* expression was significantly augmented after treatment with the unconditioned protein supplemented at the beginning of ischemia and even more when exposed to hypoxia-preconditioned α-Syn (Figure 6A) in comparison to nontreated ischemic or control cells. This latter observation suggests a toxic role of hypoxia-preconditioned α-Syn contributing to endothelial activation during ischemia. Moreover, a significant *IL-6* induction was evident on the addition of monomeric or hypoxic preconditioned α-Syn (Figure 6A). However, the expression of *IL-1*α** was comparable across the experimental groups (Figure 6A). On the contrary, treatment with recombinant α-Syn, particularly the hypoxia-preconditioned form, attenuated ischemia-induced upregulation of *VEGF-A*. Conversely, treatment with α-Syn increased the expression of *VEGFR-2* in both control and ischemic ECs. Although oxygen-glucose deprivation and reoxygenation alone did not elevate *Angpt-2* mRNA levels, α-Syn administration tended to increase *Angpt-2* expression in both control and ischemic cells. In addition, oxygen-glucose deprivation and reoxygenation immortalized human brain microvascular ECs presented a positive α-Syn staining, which was evident only after exogenous provision of hypoxic preconditioned α-Syn (Figure 6B).

## Discussion

We report a critical role of ischemic stroke-associated α-Syn accumulation in exacerbating vascular damage, impairing angiogenesis, and worsening stroke outcomes in mice. In line with previous observations showing the relevance of α-Syn in mediating ischemia-induced brain damage,^5^ we found that α-Syn null mice had longer survival and better locomotor activity in comparison to C57BL/6J mice expressing endogenous mouse α-Syn. Strikingly, we could demonstrate that acute ischemia-induced α-Syn increase and its oligomerization correlated with the expression of factors associated with endothelial activation, immune cell infiltration, basement membrane degradation and vascular permeability, and inflammation at 48 hours while reducing the expression of proangiogenic factors, angiogenesis, and tight junction protein levels at 7 days after tMCAo.

At first, we ought to see whether our model of ischemic stroke in C57BL/6J mice could increase α-Syn mRNA expression and protein levels. We found that ischemia increased α-Syn mRNA but not the levels of monomeric protein. However, we observed the accumulation of its high molecular weight species supporting the occurrence of α-Syn oligomerization, as previously observed after 72 hours of tMCAo.^26^ Nevertheless, previous studies did not identify a vascular localization of ischemia-induced α-Syn and its role in ischemia brain damage. The here-reported progressive accumulation of α-Syn at peri-vasculature of C57BL/6J ipsilateral cortex from the acute to the subacute phase of ischemia provides evidence that the vascular niche is a target of α-Syn pathology in ischemic stroke. Interestingly, in a former study, we reported that α-Syn accumulates in the perivascular space also in the postmortem brains of patients with sporadic PD,^9^ further supporting a vascular tropism of the protein’s pathological forms.

α-Syn is known as a potent chemoattractant for immune cells, including microglia in chronic neurodegenerative disorders.^27,28^ Similarly the ischemic vascular endothelium of C57BL/6J mice expresses *ICAM-1* and *MMP-9*, facilitating the recruitment and transmigration of peripheral immune cells into the injured brain.^29^ MMP-9 is released by ischemic cells including microglia^22^ and mediates degradation of basement membranes and tight junction components in the early phase of ischemic stroke,^30^ thereby facilitating peripheral immune cell entry, which in turn exacerbates the ischemic damage. On the other hand, C57BL/6S α-Syn null mice failed to induce *ICAM-1* and *MMP-9* and exhibited reduced recruitment of CD45-positive leukocytes into the ischemic cortex 48 hours after tMCAo, suggesting the absence of α-Syn may attenuate early inflammatory responses and reduce vascular damage. This was in line with a suppressed cortical expression of *HIF1*α** and its target genes involved in vascular permeability *(VEGF-A*, *VEGF-B*, *VEGFR-1*) during the acute phase after tMCAo in C57BL/6S mice that may have curtailed maladaptive vascular response that can lead to the formation of immature and leaky vessels exacerbating cerebral edema and secondary damage that influence later brain recovery as previously described.^31^ Based on previous studies that have shown that microglia are involved in vascular disintegration by physical association to brain vessels,^23^ we evaluated juxta-vascular microglia using immunofluorescence. Our data revealed a reduced association of these cells with ischemic vessels in α-Syn null mice, providing additional evidence for improved vascular protection in the absence of α-Syn. In addition, it is likely that the deposition of α-Syn around the vasculature in C57BL/6J mice promoted enhanced microglia association with vessels and hence their deleterious effect in these mice. However, despite a reduced inflammation and better vascular response, α-Syn null mice did not show a superior neuroprotection, as evidenced by comparable sensorimotor deficit and neuronal loss at 48 hours to C57BL/6J mice. This could indicate that the absence of α-Syn does not influence initial neuronal damage that results from the primary injury mechanisms activated due to hypoxia and energy depletion.^29,30^ Moreover, the suppression of initial inflammation, which led to reduced recruitment of immune cells in α-Syn null mice could paradoxically hinder the growth of collaterals that are essential to limit ischemia injury,^32^ leading to neurological deficits comparable to those of control mice. In addition, previous data indicate that reduced inflammatory response might not always lead to protection from acute ischemic stroke.^29,33^ Although, future experiments will be essential to determine whether variations in innate collateral networks between C57BL/6J and C57BL/6S mice contribute to the observed differences in stroke outcomes.

Interestingly, 7 days post-tMCAo, we observed a better survival of α-Syn null mice in line with previous findings.^5^ At this latter experimental end point, we employed elevated plus maze test to assess anxiety-like behavior and locomotor deficit as a readout of poststroke neurological impairment. In this study, sham mice showed a preference for the closed arms, which may indicate a baseline anxiety induced by the sham surgery. However, wild-type but not α-Syn null mice tMCAo mice spent more time in the open arm, suggesting an impaired anxiety response or disinhibition due to the worse ischemic brain damage in the presence of toxic α-Syn. Though the higher rate of mortality in C57BL/6J tMCAo mice might have confounded the result, α-Syn null mice also showed improved locomotor activity. Moreover, it should be mentioned that our in vivo study was conducted in a homogenous cohort of mice; thus, future studies including biological variability, like sex and age, will increase the generalizability of our findings, as sex- and age-specific responses to stroke are documented.^34^ Previous studies demonstrated that improved microvascular blood flow by newly formed vessels is associated with better functional recovery in stroke patients.^2^ Our results show that 7 days post-tMCAo, α-Syn Null mice also exhibited increased expression in proangiogenic factors (VEGF-A, VEGFR-2 and angiopoietin-2) and angiogenic activity evidenced by increased EC proliferation and higher levels of TJ proteins in the infarcted cortex relative to C57BL/6J mice. The increased proangiogenic response may have contributed to the improved recovery of α-Syn null mice, suggesting the deleterious effect of poststroke α-Syn increase and alteration on vascular plasticity and recovery that may underline phenotypic outcomes. This is in line with a recent evidence indicating that pathological accumulation of mutant α-Syn in neurons impaired neuronal activity, angiogenesis, and cerebrovascular development in young mice.^13^ It has been described that MMP-9^35^ and IL-6^36^ exert a beneficial effect in the late-phase of stroke by promoting vascular remodeling.^35^ Interestingly, we observed that 7 days after tMCAo α-Syn null mice exhibited increase in *MMP9* and *IL-6* expression that could thus justify the enhanced vascular recovery in these animals, when compared with C57BL/6J mice. This is also in agreement with the mild concomitant reduction in TIMP1 expression, a protein involved in the regulation of the activity of MMPs at posttranslational level, observed in the α-Syn Null mice.

It is worth mentioning that C57BL/6S α-Syn null mice also exhibit a spontaneous deletion of MMRN1 (multimerin 1), a protein expressed by platelets and ECs and involved in platelet adhesion and aggregation.^15^ At variance, others have reported that platelets of α-Syn knockout mice had higher CD62P (cluster of differentiation 62P; P-selectin) expression, activation, and degranulation, whose effect would lead to increased thrombus formation and worsened ischemic injury.^7^ However, the contribution of platelets to stroke outcomes in our C57BL/6S α-Syn null mice is difficult to predict, as their behavior may result from a complex interplay between the loss of MMRN1 and α-Syn. Hence, future studies are needed to dissect the individual effects of these proteins.

Because the expression of α-Syn in ECs is very low,^9^ it is possible that ischemic neuronal and other non-neuronal cells are the main sources of α-Syn after stroke. Though the exact function of physiological α-Syn in non-neuronal cells still needs to be fully disclosed, studies have shown that under basal conditions the protein is essential for maintaining vascular endothelial function, while reduction in α-Syn level is associated with impaired vascular function during aging.^37^ On the other hand, stimulation of ECs with toxic α-Syn protofibrils impaired endothelial function.^9^ Thus, we hypothesized that extracellular α-Syn might modulate EC function during ischemia. The preconditioning of recombinant monomeric α-Syn in 18 hours of hypoxia and supplementation to ECs resulted in the uptake and accumulation of the protein inside the cells, as evidenced by clumps on immunofluorescence. In this latter experimental condition, in line with our in vivo data, we observed significant alterations of vascular markers such as *ICAM-1*, *IL-6*, *VEGF-A*, *VEGFR-2*, and *Angpt-2*. This observation suggests the direct effect of the protein on the EC inflammatory profile and response to ischemic stimulus.

Overall, our data suggests that a reduction in early vascular damage together with a heightened delayed beneficial angiogenesis in α-Syn null mice, underlies the observed improved functional recovery and higher survival rates in these mice during the later phases after ischemia. Indeed, the higher levels of tight junction proteins and increased vascular volume in mice without α-Syn may evidence the formation of stable, functional blood vessels seen which can improve tissue perfusion and recovery. Overall, these data suggest that ischemia-induced α-Syn influences brain damage in a temporally dynamic manner where its increased accumulation in the peri-vasculature over time may exacerbate toxicity and worsen outcomes beyond the acute phase in C57BL/6J mice. Thus, aggregated α-Syn stands as a new target for cerebrovascular protection after ischemic stroke. α-Syn pathology is a major culprit in PD, but the exact pathological process that triggers α-Syn alterations and subsequent contribution to neuronal loss is still debated. As such, a challenge remains whether, when, and how to target α-Syn pathology. On the other hand, studies from others and ours have shown that ischemia-reperfusion injury induces changes in the protein that contribute to the loss of its physiological role with subsequent formation of toxic aggregates. We think that toxic α-Syn species formed after ischemic stroke can be targeted during the early phase of ischemic stroke with already existing monoclonal antibodies, enhancing their clearance, thereby mitigating aggregation-led sequestering of the functional protein that may render it unavailable for reparative processes or basal vascular maintenance. This is especially relevant as ischemic stroke patients have a higher risk for developing PD.^38^

## Article Information

### Acknowledgments

Endotoxin-free monomers of human recombinant α-Syn (alpha-synuclein) was kindly donated by Professor Isabella Russo, University of Brescia, Italy. All authors agreed on the content of this manuscript before its submission.

### Sources of Funding

This work was supported by the European Union’s Horizon 2020 research and innovation program under the Marie Skłodowska-Curie grant agreement no. 813294 and by Fondazione Roche as part of the seventh edition of the Roche per la Ricerca Indipendente grant to Dr Bogale. A. Bellucci is grateful to the Michael J Fox Foundation for Parkinson’s Research, NY, Target Advancement Program, grant ID no. 10742.01.

### Disclosures

None.

### Supplemental Material

Supplemental Methods

Tables S1–S2

Figure S1–S3

ARRIVE Guidelines

## Supplementary Material
