# Supplementary material for: Experimental Ischemic Stroke–Induced Alpha-Synuclein Pathology Enhances Endothelial Inflammatory Response and Impairs Angiogenesis
Source: Stroke. 2025 Sep 18;56(12):3424–37. doi: 10.1161/STROKEAHA.125.052265 (PMC12643574; doi:10.1161/STROKEAHA.125.052265)
Supplement: Supplementary file 1 [file str-56-3424-s001.pdf]

## SUPPLEMENTAL MATERIAL

### Detailed methods

Please see the Major Resources Table in the Supplemental Materials.

Mouse Experiments

All animal experiments were approved by ethical committee at the Istituto di Ricerche Farmacologiche Mario Negri IRCCS and from the Italian Ministry of Health (authorization number n°383/2021-PR) and performed in compliance with national and international guidelines for animal care and use. Male 9-to-12 weeks old C57BL/6J wild type (WT, Charles Rivers-Italy) and C57BL/6JOlaHsd (C57BL/6S, Envigo/Inovit) were used. The C57BL/6S mice are subpopulations of C57BL/6J inbred mice that exhibit a spontaneous deletion of the *SNCA* (*Synuclein Alpha) and* Mnrn1 (*multimerin 1*) gene locus.[14,15] All the protocols and details of this study are in accordance with the ARRIVE guideline as previously described.[16] We used only male mice since estrogen affect ischemic outcome in experimental models, which could interfere with interpretation of the results.[17]

Experimental design

Animals were randomly assigned to sham and tMCAo groups with different reperfusion duration using a lottery-drawing box. All biochemical (PCR, and Western blot analysis) and histological (immunohistochemical staining and quantifications) were performed by investigators who were blinded to experimental group assignments. Given the explorative nature of our research, we defined the total numerosity of ischemic mice based on a power test study considering a decrease of 30% of neuroscore at 48h as an index of ameliorated sensorimotor deficits. Group size was defined pre hoc using the formula: $n= 2\sigma^2 f(\alpha,\beta) / \Delta^2$ (SD in groups = $\sigma$, type 1 error $\alpha= 0.05$, type II error $\beta= 0.2$, percentage difference between groups $\Delta= 30$). For each measure, the standard deviation (SD) between groups was calculated on the basis of the neuroscore measured on WT mice in previous experiments using the same model ($\sigma= 25$, yielding $n= 10.9$). At each experimental endpoint, the mice were divided into two groups for tissue collection, facilitating cross-sectional histological or molecular analysis.

Focal cerebral ischemia

Mice were anesthetized with 3% and maintained by 1.5% isoflurane inhalation in a 70% $N_2O$/ 30% $O_2$ mixture under spontaneous breathing condition. Focal cerebral ischemia was produced by intraluminal occlusion of the right middle cerebral artery (MCA) for 60 minutes (tMCAo) as described previously. [18] Rectal temperature was kept at 37°C by a heating pad during surgery and during MCA occlusion. During the MCA occlusion, mice were monitored for intra-ischemic deficits (see below inclusion/exclusion criteria). Total of 72 mice (12 sham-operated and 60 ischemic mice) were used in this study, including 1 mouse that was excluded from further assessments because of major surgical violation during ischemic induction.

Inclusion/exclusion criteria

Mice were included in the study if ischemia is induced successfully, i.e. filament is correctly positioned in the MCA. Thus, ischemic animals were included if presenting ≥3 of the following intra-ischemic deficits:

1. The palpebral fissure had an ellipsoidal shape (not the normal circular one)
2. One or both ears extended laterally
3. Asymmetric body bending on the ischemic side
4. Limbs extended laterally and did not align to the body.

Mice were excluded if:

1. They died during MCA surgery.
2. There was a major surgical violation.

3. They had a decrease in body weight < 35% before sacrifice compared to baseline

Health monitoring

The surgical procedure was performed on mice under general anesthesia. During awakening after surgery and over the next 48h leading up to the sacrifice, ischemic animals exhibited common deficits associated with this model. These deficits were meticulously monitored in accordance with the IMPROVE guidelines for ischemic animals. [19] If severe deficits and pain occurred, we followed an analgesia protocol (administering Buprenorphine subcutaneously at a dose of 0.05-0.1 mg/kg every 8-12 hours) or euthanizing mice before reaching the experimental endpoint and assigning them the worst behavioral score.

Behavioral deficits

Forty-eight hours after tMCAo, each mouse underwent neuroscore evaluation for assessment of sensorimotor deficits. Scores ranged from 0 (healthy) to 56 (worst performance) combining general and focal deficits. The general deficits describe the general well-being of the mouse with a score between 0 and 28. This score includes information on the physical appearance of the mouse such as: fur (0-2), ears (0-2), eyes (0-4), posture (0-4), spontaneous activity (0-4) and presence of epileptic seizures (0-12). Focal deficits describe neurological damage with a score between 0 and 28 and were evaluated through observations on body symmetry (0-4), gait (0-4), ability to climb a 45° inclined plane (0-4), spontaneous circling behavior (0-4), forelimb symmetry (0-4), compulsory circling (0-4) and whisker response (0-4).

Elevated Plus Maze (EPM)

The elevated plus maze was used to evaluate anxiety-like behavior in a new environment as a measure of cognitive deficit at 7 days after tMCAo. The maze consists of two opposing open arms (50 cm × 10 cm), an open platform (10 cm × 10 cm) in the center, and two opposing closed arms (50 cm × 10 cm). The maze was elevated 50 cm from the floor. At the beginning of the test, the mice were placed on the central platform facing one of the open arms. The cumulative time spent in the open and closed arms and distance travelled in the entire platform was recorded during a 5 min period using Noldus Etho Vision XT video tracking software. The maze was cleaned with 70% ethanol between each test.

Tissue processing for histology and immunofluorescence

Mice were transcardially perfused with 30 mL of phosphate buffer (PBS) 0.1 mol/L, pH 7.4, followed by 60 mL of chilled paraformaldehyde (4%) in PBS. The brains were carefully removed from the skull and post-fixed for 6 h at 4°C, then transferred to 30% sucrose in 0.1 mol/L PBS for 24 h until equilibration. The brains were frozen by immersion in isopentane at – 45°C for 3 min and stored at − 80°C until use. 48h and 7d after tMCAo, mice were deeply anesthesized with intraperitoneal (i.p) injection of ketamine and medetomidine (100 mg/ml ketamine; 1 mg/ml medetomidine; 300 µl/mouse). Mice were transcardially perfused with 30 ml of PBS, 0.1 mol/liter, pH 7.4, followed by 60 ml of cold paraformaldehyde (4%) in PBS.

Immunohistochemistry

To analyze the infiltration of peripheral immune cells into ischemic brain, we performed immunohistochemistry of leukocytes with anti-mouse-cluster differentiation (CD) 45 (1:50, BD Biosciences Pharmigen, San Jose, CA) on 20 µm-thick coronal brain sections that were cut serially at 320 µm intervals. Briefly, Sections were washed with PBS, dried for an hour, and blocked with 10% FBS for 1h at room temperature, followed by incubation with anti-mouse-CD45 overnight at 4°C. After blocking endogenous peroxidase, sections were incubated with anti-rat biotinylated secondary antibody (Vector laboratories, CA, USA) for 1h at RT. Immunoreactivity was visualized by the avidin-biotin complex (Vector) method. Positive cells were stained by reaction with 3, 3 diaminobenzidine tetrahydrochloride (DAB, Vector Laboratories, CA, USA) and mounted on coverslips. Images were captured on an Olympus BX61 Virtual Stage microscope at 20× magnification. CD45 positive cells were counted using the ImageJ cell counter plugin and expressed as the number of cells per total staining area (number per mm2) for subsequent statistical analysis.

Immunofluorescence

To analyze the cellular and perivascular localization of α-Syn, cryosections were fixed in methanol 20%, blocked with 3% normal goat serum for 1h, and incubated overnight at 4 °C with the anti-α-Syn (BD biosciences #610787), anti-cluster differentiation (CD)31 (1:50, BD biosciences, #550274), anti-PSD-95 (1:200, Abcam, #ab18258). To analyze microglia-contact with vessels, sections were labeled with anti-Iba1 (1:1000, Wako, #019-19741) and anti-CD31. To evaluate angiogenesis, sections were marked with CD31 and a cell proliferation marker, Ki67 (1:400, Cell signaling, #D3B5). Then, sections were incubated with secondary antibody conjugated to Alexa-fluoro 488, or 647 or 555 for 1h at room temperature. For *in vitro* experiment, cells were washed with pbs, fixed in 4% PFA. Nuclei were visualized using DAPI counterstains. Immunofluorescence images were acquired at 40x or 60x magnification by a Nikon A1 confocal system and captured using NIS-Elements software. Images were then re-constructed using Imaris software.

Western blot analysis

Total proteins from brain tissues were extracted with a tissue homogenization buffer made up of 1M Tris-HCl (pH 7.4), sterile DEPC water, sucrose, 0.5mM EDTA (pH 8), and 0.5mM EGTA (pH 7) complete protease and phosphatase inhibitor mixture. Protein concentration in the samples was measured by using the Bio-Rad protein assay kit. 15-30μg of proteins were run on 8%-12% polyacrylamide gels, transferred onto a polyvinylidene fluoride (PVDF) membrane and probed with primary antibodies: anti-mouse α-Syn (1:1000, BD biosciences, #610787,), anti-mouse actin (1:10,000, Merck, MAB1501), anti-occludin (1:1000, Thermo fisher, #71-1500,), and anti-zonulin1 (1:1000, Thermo fisher, #33-9100). Secondary antibodies used were goat anti-mouse and anti-rabbit HRP. Densitometry analysis of the bands was performed using ImageJ software. All bands were normalized to actin levels as a control of equal loading of samples in the total protein extracts, and the resulting data were subjected to statistical analysis.

In vitro ischemia

The ihBMECs cell line was grown in fibronectin-coated ibidi 96-well micro plates to allow adhesion and growth with MCDB (Biowest serum-reduced supplementation medium) supplemented with 0.1% Hydrocortisone and 1% ECG (Endothelial Cell Growth Factor), Fetal Bovine Serum (FBS) 5%, L-Glutamine 1%, Penicillin / Streptomycin 1% at 37 °C in a humidified incubator with 5% CO2. On the day of the experiment, cells in control plate were incubated with low-glucose DMEM with sodium bicarbonate and sodium pyruvate, without L-glutamine and no FBS at 37 °C in a humidified incubator with 5% CO2. In contrast, cells in OGD plate were bathed with DMEM, no glucose, no glutamine, and no FBS in an OGD chamber that was continuously infused with a gas mixture containing 99% nitrogen and <1% oxygen at 37°C for 2h. For reperfusion experiment, half medium was changed to low-glucose DMEM and transferred to a humidified incubator with 5% CO2 for 4h. To analyze the direct effects of α-Syn on ihBMECs subjected to OGD-Reperfusion, cells were treated with non-hypoxia preconditioned or hypoxia preconditioned 6ug/ml (0.415uM) endotoxin-free monomers of human recombinant α-Syn. At the end of experiment, cells from most wells were scrapped off for subsequent RNA extraction (see below) while other wells were PFA fixed for IF. For the latter case, cells were fixed in 4% PFA for 15 minutes, washed twice with PBS and then incubated with primary antibodies: anti-α-Syn (BD biosciences, #610787) and Phalloidin-ifluor-488, (1:20, Abcam, ab176753).

Quantitative Real-time -PCR (qRT-PCR)

RNA was extracted from ipsilateral hemispheres using the RNeasy Mini Kit for total RNA extractions (PureLink™ RNA Mini Kit, thermos fisher). Then, 1 μg of total RNA was retro transcribed to cDNA. cDNA (1-5 μL) was amplified with Power SYBR Green (Applied Biosystems) and a mix of forward and reverse primers (10 μM) and relative gene expression determined with ΔΔCt method. For normalization of quantification, β-actin was used as housekeeping gene. Primer sequences are detailed in Supplementary Information.

Statistics

Group comparisons were conducted using t-tests, Mann-Whitney tests, or relevant two-way analysis of variance (ANOVA) followed by the appropriate post hoc test. Bartlett's test checked equal variances and, if not equal, a Welch's correction was applied to the test. Statistical analyses were performed using GraphPad Prism (GraphPad Software Inc., San Diego, CA, USA, version 9.0). All data were presented as mean and standard deviation (SD). *P*-values lower than 0.05 were considered statistically significant.

**Supplementary Table 1.** All C57BL/6J and C57BL/6JOlaHsd mice that were subjected to tMCAo showed a ≥3 intraischemic deficits score, thereby meeting the inclusion criteria (successful MCA occlusion), with the exception of one WT mouse that was sacrificed due to a major surgical violation.

| **Mouse strain** | **Time of sacrifice (after reperfusion)** | **No. of intraischemic deficits (No. of mice)** | **Major surgical violation (No. of Mice)** |
|---|---|---|---|
| C57BL6/J (WT) | 48h | 4 (n= 6) | n= 1 |
| C57BL/6JOlaHsd (a-syn null) | 48h | 4 (n= 6) | |
| C57BL6/J (WT) | 7d | 4 (n= 6) | |
| C57BL/6JOlaHsd (a-syn null) | 7d | 4 (n= 6) | |

**Slice selection and image acquisition**

Three brain coronal sections per mouse at +1.54, +0.50 and – 0.94 mm from bregma, were used to quantify neuronal cells and immunostainings. The entire brain sections were acquired at 20X by an Olympus BX-61 Virtual Stage microscope, with a pixel size of 0.346 mm. Acquisition was done over 10 μm thick stacks, with a step size of 2 μm. The different focal planes were merged into a single stack by mean intensity projection to ensure consistent focus throughout the sample.

**Definition of regions of interest and image quantification**

Images were analyzed using Fiji software. The anatomical regions affected by MCA occlusion were the cerebral cortex, the striatum and hippocampus. For neuronal count at 48h and 7d after tMCAo, the regions of interest were placed in the lesioned area (cresyl violet pale staining) in the ipsilateral cortex and in the respective contralateral cortical region as depicted in Fig. 1e and f Neuronal count was performed by segmentating the cells and excluding the round-shaped signal sized below the area threshold of 25 μm2 that is known to be associated with glial cells as reported previously (ref). For immunostaining quantifications the cortical and striatal regions of interest were placed in the ipsilateral cortex involved in the ischemic lesion. For CD-31, Ki-67 and Iba1, all yielding a sharp signal-to-noise ratio, the positive signal was segmentated by applying a gray-level threshold cuttingoff the background. Immunostained areas were expressed as positive pixels/total assessed pixels and reported as the percentage of total stained area as previously described (ref) . CD45-positive cells had two morphologies: a leukocyte-like shape corresponding to cells with a rounded body without branches and high expression of CD45 (CD45high) and a microglia-like shape with a small body and several branches and fainter expression of CD45 (CD45low). Quantification was done on CD45high cells after proper segmentation of the positive signal. CD45high counts were reported as density of cells/mm2.

Primers were designed to span exon junctions in order to amplify only spliced RNA using PRIMER-3 software (http://frodo.wi.mit.edu/) based on GenBank accession numbers. Basal expression of genes selected for analysis in various brain cells was computed as shown below (figure 11) from the mouse single-cell BrainRNAseq online database (https://www.brainrnaseq.org/). Arrow indicates the cells primarily expressing these genes.

**Supplementary Table 2.** List of primers used

| *Gene* | *Forward primer* | *Reverse primer* |
|---|---|---|
| *hβ-actin* | *CCAGCTCACCATGGATGATG* | *ATGCCGGAGCCGTTGTC* |
| *mActin* | *CGCGAGCACAGCTTCTTT* | *GCAGCGATATCGTCATCCAT* |
| *mAngpt2* | *ACCAGACAGCAGCACAAACT* | *GAAATAGAATGTTGGAGAAGCTGCA* |
| *mHIF1α* | *GGAAATGAGAGAAATGCTTACACACA* | *AGGGTGCACTTCATTCTGAGA* |
| *mICAM-1* | *CTGTGCTTTGAGAACTGTGGC* | *GCTCCACACTCTCCGGAAAC* |
| *hICAM1* | *TGATGGGCAGTCAACAGCTA* | *GGTAAGGTTCTTGCCCACTGG* |
| *hVEGF-A* | ACTGCCATCCAATCGAGACC | TATGTGCTGGCCTTGGTGAG |
| *hVEGFR-2* | CTGGACTCTCTCTGCCTACCT | AGAACCATACCACTGTCCGTC |
| *hAngpt2* | GGACCCCACTGTTGCTAAAGA | CCATCCTCACGTCGCTGAAT |
| *mIl-6* | *CCTACCCCAATTTCCAATGCT* | *TATTTTCTGACCACAGTGAGGAAT* |
| *hIl-6* | *ACAACCTGAACCTTCCAAAG* | *ACCTCAAACTCCAAAAGACC* |
| *hil-1a* | *TGAAGAAGACAGTTCCTCCATTG* | *CTTCATGGAGTGGGCCATAG* |
| *mMMP9* | *CGTCATTCGCGTGGATAAGG* | *CCATGGCAGAAATAGGCTTTGT* |
| *mTIMP1* | *TCAAAGACCTATAGTGCTGGCTG* | *AGTGTCACTCTCCAGTTTGCA* |
| *mSNCA* | *GGCCAAGGAGGGAGTTGT* | *GCTCCCTCCACTGTCTTCTG* |
| *mVEGF-A* | *AACGATGAAGCCCTGGAGTG* | *GCTGGCTTTGGTGAGGTTTG* |
| *mVEGF-B* | *AGAGCTCAACCCAGACACCT* | *GTGAAGCAGGGCCATAAAAGC* |
| *mVEGFR-1* | *GGATGAGGGTGTCTATAGGTGC* | *CAGGTTTGACTTGTCTGAGGTTC* |
| *mVEGFR-2* | *TGGTCAAACAGCTCATCATCCT* | *CGCCAATGGTTGTTGTCTGA* |

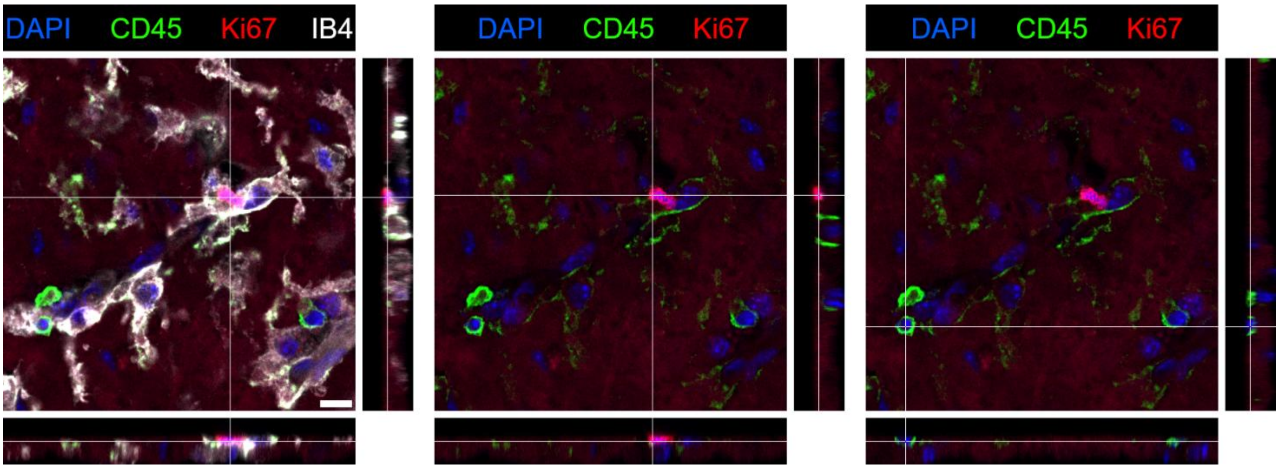


**Fig. S1.** Left panel) Representative immunofluorescence microphotographs of the ischemic cortex labelled for CD45 (green), Ki67 (red), blood vessels (labelled by isolectin B4, IB4, white) and nuclei (labelled with DAPI, blue) at 7d after tMCAo, scale bars 10 µm. Middle panel) A Ki67 positive cell associated with the blood vessel was negative for CD45. Right panel) Cells highly expressing CD45 and located nearby the blood vessels, identified as infiltrating leukocytes, were negative for Ki67.

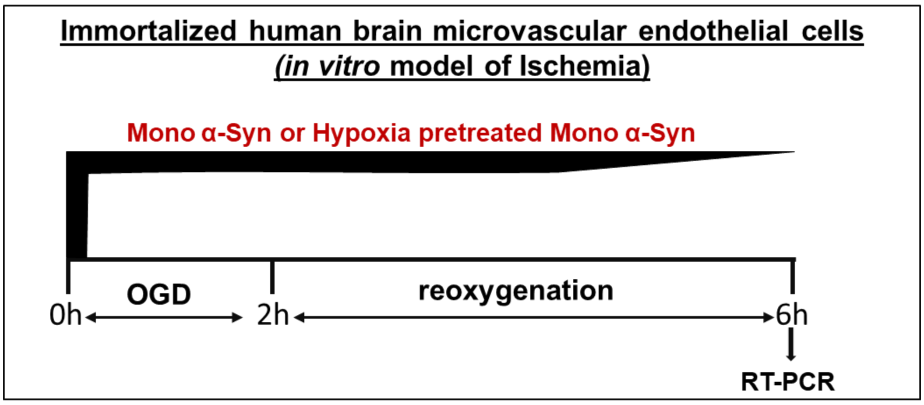


**Fig S2.** Schematic diagram of the experimental plan. Briefly, ihBMECs were exposed to 2h OGD followed by 4h reoxygenation (R) in the presence or absence of recombinant monomeric or hypoxia pretreated α-Syn (added at the beginning of OGD).

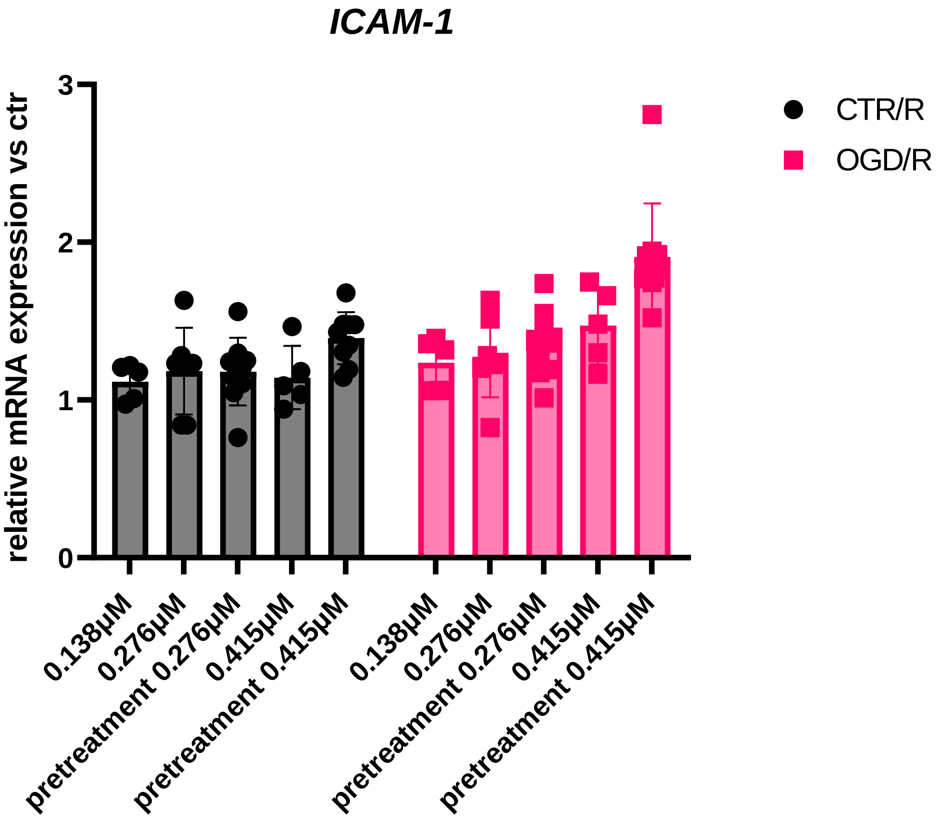


**Fig S3.** Preliminary data of *ICAM-1* mRNA expression in ihBMECs after 2 (0.138µM), 4 (0.276µM) and 6µg/ml (0.415µM) of naïve or hypoxia pretreated monomeric alpha-synuclein supplied at the beginning of OGD and reoxygenation experiment.

**ARRIVE GUIDELINES checklist**

# The ARRIVE guidelines 2.0: author checklist

## The ARRIVE Essential 10

These items are the basic minimum to include in a manuscript. Without this information, readers and reviewers cannot assess the reliability of the findings.

| Item | | Recommendation | Section/line number, or reason for not reporting |
|---|---|---|---|
| **Study design** | 1 | For each experiment, provide brief details of study design including: | Figure legends |
| | | a. The groups being compared, including control groups. If no control group has been used, the rationale should be stated. | |
| | | b. The experimental unit (e.g. a single animal, litter, or cage of animals). | Methods and detailed methods |
| **Sample size** | 2 | a. Specify the exact number of experimental units allocated to each group, and the total number in each experiment. Also indicate the total number of animals used. | Methods and detailed methods |
| | | b. Explain how the sample size was decided. Provide details of any *a priori* sample size calculation, if done. | Methods and detailed methods |
| **Inclusion and exclusion criteria** | 3 | a. Describe any criteria used for including and excluding animals (or experimental units) during the experiment, and data points during the analysis. Specify if these criteria were established *a priori*. If no criteria were set, state this explicitly. | Methods |
| | | b. For each experimental group, report any animals, experimental units or data points not included in the analysis and explain why. If there were no exclusions, state so. | Methods |
| | | c. For each analysis, report the exact value of *n* in each experimental group. | Figure legends |
| **Randomisation** | 4 | a. State whether randomisation was used to allocate experimental units to control and treatment groups. If done, provide the method used to generate the randomisation sequence. | Methods |
| | | b. Describe the strategy used to minimise potential confounders such as the order of treatments and measurements, or animal/cage location. If confounders were not controlled, state this explicitly. | Methods |
| **Blinding** | 5 | Describe who was aware of the group allocation at the different stages of the experiment (during the allocation, the conduct of the experiment, the outcome assessment, and the data analysis). | |
| **Outcome measures** | 6 | a. Clearly define all outcome measures assessed (e.g. cell death, molecular markers, or behavioural changes). | Methods and detailed methods |
| | | b. For hypothesis-testing studies, specify the primary outcome measure, i.e. the outcome measure that was used to determine the sample size. | Methods |
| **Statistical methods** | 7 | a. Provide details of the statistical methods used for each analysis, including software used. | Figure legends |
| | | b. Describe any methods used to assess whether the data met the assumptions of the statistical approach, and what was done if the assumptions were not met. | |
| **Experimental animals** | 8 | a. Provide species-appropriate details of the animals used, including species, strain and substrain, sex, age or developmental stage, and, if relevant, weight. | Methods and detailed methods |
| | | b. Provide further relevant information on the provenance of animals, health/immune status, genetic modification status, genotype, and any previous procedures. | Methods and detailed methods |
| **Experimental procedures** | 9 | For each experimental group, including controls, describe the procedures in enough detail to allow others to replicate them, including: | Methods and detailed methods |
| | | a. What was done, how it was done and what was used. | |
| | | b. When and how often. | |
| | | c. Where (including detail of any acclimatisation periods). | |
| | | d. Why (provide rationale for procedures). | |
| **Results** | 10 | For each experiment conducted, including independent replications, report: | Figure legends |
| | | a. Summary/descriptive statistics for each experimental group, with a measure of variability where applicable (e.g. mean and SD, or median and range). | |
| | | b. If applicable, the effect size with a confidence interval. | |
